# Machine learning approaches to predicting no-shows in pediatric medical appointment

**DOI:** 10.1038/s41746-022-00594-w

**Published:** 2022-04-20

**Authors:** Dianbo Liu, Won-Yong Shin, Eli Sprecher, Kathleen Conroy, Omar Santiago, Gal Wachtel, Mauricio Santillana

**Affiliations:** 1 Boston Children’s Hospital, Boston, MA USA; 2 Department of Pediatrics, Harvard Medical School, Boston, MA USA; 3 School of Mathematics and Computing (Computational Science and Engineering), Yonsei University, Seoul, Republic of Korea; 4 Department of Epidemiology, Harvard T.H. Chan School of Public Health, Boston, MA USA

**Keywords:** Translational research, Health services

## Abstract

Patients’ no-shows, scheduled but unattended medical appointments, have a direct negative impact on patients’ health, due to discontinuity of treatment and late presentation to care. They also lead to inefficient use of medical resources in hospitals and clinics. The ability to predict a likely no-show in advance could enable the design and implementation of interventions to reduce the risk of it happening, thus improving patients’ care and clinical resource allocation. In this study, we develop a new interpretable deep learning-based approach for predicting the risk of no-shows at the time when a medical appointment is first scheduled. The retrospective study was conducted in an academic pediatric teaching hospital with a 20% no-show rate. Our approach tackles several challenges in the design of a predictive model by (1) adopting a data imputation method for patients with missing information in their records (77% of the population), (2) exploiting local weather information to improve predictive accuracy, and (3) developing an interpretable approach that explains how a prediction is made for each individual patient. Our proposed neural network-based and logistic regression-based methods outperformed persistence baselines. In an unobserved set of patients, our method correctly identified 83% of no-shows at the time of scheduling and led to a false alert rate less than 17%. Our method is capable of producing meaningful predictions even when some information in a patient’s records is missing. We find that patients’ past no-show record is the strongest predictor. Finally, we discuss several potential interventions to reduce no-shows, such as scheduling appointments of high-risk patients at off-peak times, which can serve as starting point for further studies on no-show interventions.

## Introduction

Scheduled but unattended medical appointments, often referred to as “no-shows,” have direct negative impacts on patients’ health and hospital and clinics’ resource utilization^1,2^. Different healthcare systems experience no-show rates that range between 3% and 80%. As continuity of treatment, preventive services, and medical check-ups cannot be delivered when a patient misses an appointment, no-shows at appointments have been associated with poor control of chronic diseases and delayed presentation to care^3,4^.

There are different reasons for no-shows that include scheduling problems, time conflicts (e.g., with patient’s work schedule), and traffic and environmental factors^5–9^. Previous studies have shown that patients with lower socioeconomic status, prior history of no-shows, and public insurance are more likely to miss their medical appointments^9–12^.

Although there have been multiple attempts to predict medical appointment no-shows based on statistical and machine learning strategies, the recent availability of large amounts of recorded data in electronic health records and advances in deep learning have enabled us to conduct more personalized prediction of no-show risks^13–17^. Underexplored challenges in the prediction process still remain despite these efforts. In this study, we tackle several challenges in no-show predictions that have not been studied in the literature. Our main contributions are four-fold: we propose an explicit methodology for how to handle missing data, we leverage local weather information, and we propose ways to improve interpretability as an essential part of the modeling exercise. We discuss potential interventions that can be implemented by hospitals and clinics with limited resources to prevent no-shows based on actionable items identified in our analyses. We address each of these as follows.

Missing data are very common in healthcare systems due to diverse factors such as patient privacy, patient willingness to share their information, and hospital operational reasons, where the missing pattern can be either random or non-random^18–20^. In our approach, we develop two different machine learning strategies that allow us to include records of patients with multiple missing fields of information in the training process. Our methods thus allow us to estimate the likelihood of a no-show for unseen patients even in the presence of missing information. This is done by using two complementary strategies, one via supervised learning data imputation techniques and a second one using missingness-type indicators. Our proposed methodology is flexible and adaptable to the likely different patterns of missing data in other hospitals and clinics.

The rates and reasons for no-shows may differ by hospitals, departments, and even healthcare providers. For example, some hospitals have patient populations that rely heavily on public transportation to get to their medical appointments and, that in turn, are often more vulnerable to abrupt changes in weather. In our research, we explore the impact that local weather information may have on patterns of “no-show”.

Thirdly, in healthcare, deep learning-aided black box solutions are not easily adopted by medical staff even if they have high predictive performance^21^. In this study, we interpret the trained machine learning model in such a way that the importance of each predictor in predicting no-show risks of each patient can be precisely estimated. This will help medical staff understand how each model works.

Finally, various interventions such as phone calls and reminder messages have been attempted in medical systems to mitigate no-shows^3,12,22^. However, these intervention approaches are not always available to community clinics or in developing countries. As an alternative, we estimate the effects of some potential interventions that may be easy to implement such as scheduling appointments for patients showing a higher risk of no-shows on a day with likely better weather conditions for example. Other strategies that can be employed include providing patients with transportation means on a day with bad weather or providing home visits, or telemedicine consults, for patients with high risks of no show.

## Results

We retrospectively collected records from 161,822 hospital appointments made by 19,450 patients between January 10th, 2015 and September 9th, 2016 at Boston Children’s Hospital’s primary care pediatric clinic. Patient-related information, including age, gender, previous no-show rate, insurance type, etc., was used as input to train a machine learning model aimed at predicting the risk of a patient’s no-show at the time when the appointment was scheduled. Given how common patients’ records contained missing information (about 77% of the records) in our data, we designed methods that are capable of handling this via data missingness indicators strategies (refer to the Method subsection for details). As a reference, to motivate the need for a more sophisticated modeling technique to tackle this problem, we also include the predictive performance results of a simple logistic regression approach in the Supplementary materials. In order for our methods to capture the potential non-linear relationships among different features and our desired outcome, we used a neural network approach. Predictive performance of our model was evaluated in a collection of observations not used to train our models, that is, in a strictly out-of-sample fashion.

To evaluate performance of different approaches, we conducted a series of experiments and analyses:

1. We trained a machine learning model on only appointment records without missing information and tested the performance on out-of-sample records without missing information.
2. Using supervised missing data imputation techniques or missingness-type indicators, we trained predictive models on all records -including those with missing information and records without missing information. Performance of models obtained were tested on (a) only out-of-sample records without missing information, (b) only out-of-sample records with missing information and c) out-of-sample records with missing information and out-of-sample records without missing information.
3. We explored if including local weather information as input features will improve accuracy of the predictive models
4. We analyzed factors associated with no shows and suggested potential actionable items to avoid no shows.

**Fig. 1:**
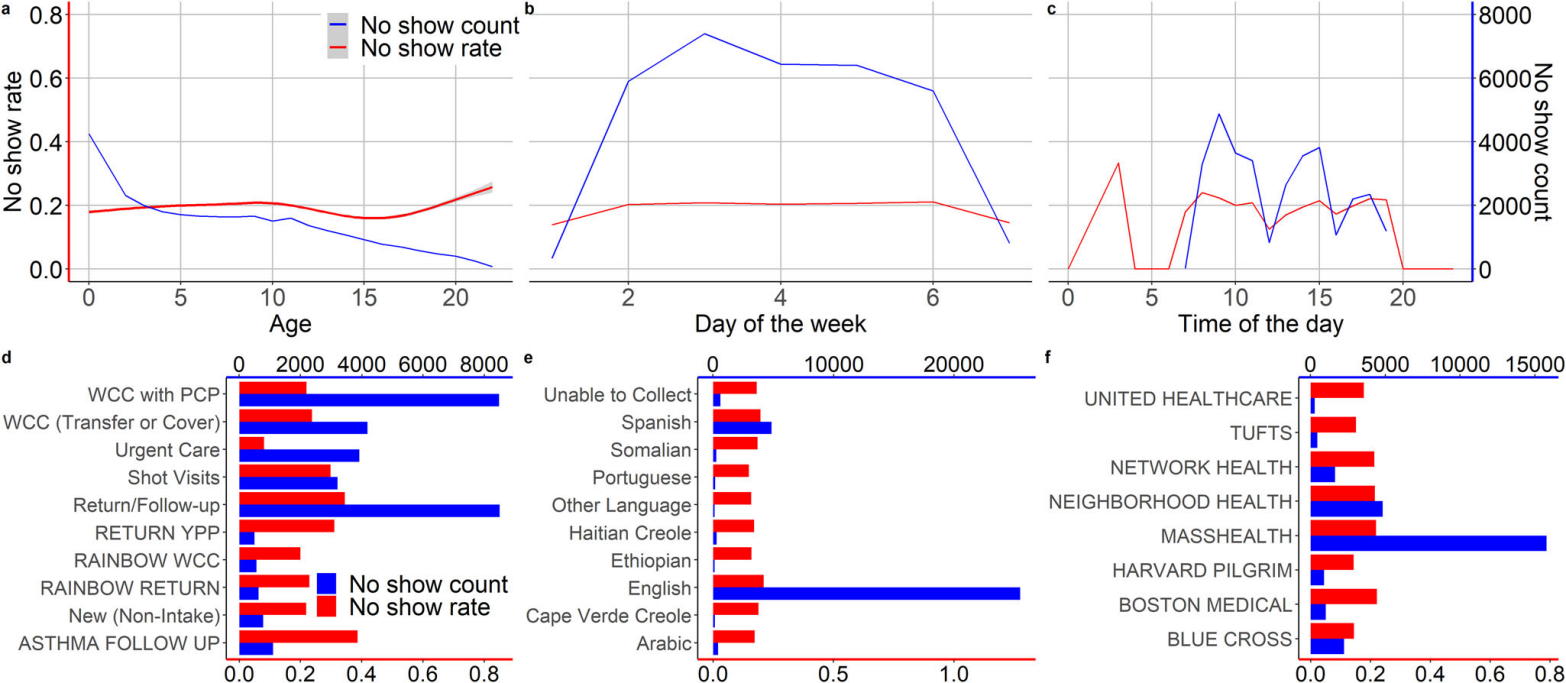
Summary statistics of no-show rates and counts. **a** Distribution of medical appointment no-show rates and counts over ages at a pediatric primary care clinic in a major academic Children’s Hospital in the U.S. (patients above age 18 were excluded from our analysis). **b** Distribution of no-show rates and counts over days of the week. **c** Distribution of no-show rates and counts over hours of day. **d** Distribution of no-show rates and counts over visit types. **e** Distribution of no-show rates and counts over spoken languages (languages were excluded from the predictive model training). **f** Distribution of no-show rates and counts over health insurance types.

In the cohort, patients’ ages ranged between 0 and 18 years in our dataset (Mean = 6.4 and SD = 5.6) were included into the analyses. The studied dataset includes the medical record number (MRN) of each patient, appointment date and time, appointment status (including completed, canceled, and no-show cases), demographic and health insurance coverage information for each patient, information on information about historical appointments, information about the healthcare provider, and health insurance plans (refer to List 1 and Supplementary List 1). According to the record in Table 1, patients did not keep 20.3% of their appointments. Distributions of no-shows counts, no show rates which we define as percentage of appointments where the patients neither showed up nor canceled, days of the week, types of appointment, spoken languages by the patient’s family, and types of health insurances are shown in Fig. 1

**Table 1:** Study cohort (appointment status).

Appointment status	Visit count	Patient count
Canceled	6633	4840
Completed	122,277	18,264
No-shows	32,912	12,749

*Fairness.* The goal of this project is to develop a methodology that benefits all patients regardless of social vulnerabilities such as racial and ethnic backgrounds. It is clear that the inclusion of race and race proxies (language, for example) as inputs in no-show prediction models may lead to unexpected and undesirable racial disparities. For example, if a likely no-show (as predicted by a model) is used to overbook physicians’ schedules, the socially vulnerable groups associated with the highest no-show risk may be scheduled in undesirable time slots that may consequentially lead to longer waiting times or inconvenient transportation schedules^23,24^. Therefore, in order to minimize the emergence of potentially unfair outcomes, we explored excluding all race and race proxy features from our proposed no-show prediction models. These race and race proxy features include: race, language, and ethnicity of the patient, whether the patient is Hispanic or not and whether the patient needs an Interpreter. Our empirical results show that exclusion of the race-related features does not cause a statistically significant decrease in the predictive model performance (*p*-value > 0.1, refer to Supplementary Fig. 1). Therefore, unless otherwise mentioned, race and race proxy features are excluded from predictive model training in all subsequent sections but are kept in associated factor analyses.

### Performance of predictions of no-shows with complete information of patents (i.e., no missing data)

As an initial step and as a way to potentially measure the “best-case scenario” in our predictive study, we built a predictive modeling approach in a population of patients for whom all fields of information were available. As mentioned above, only 23% of medical appointments were scheduled with full information available. Thus, all the features available in the dataset (shown in List 1) were utilized to build our predictive model. A multi-layer artificial neural network model was employed to solve the binary classification problem of determining whether a patient would or would not show up in a scheduled appointment time slot. Details of the architecture and training process of the machine learning model can be found in the “Materials and Methods” section. In the first stage of the study, records with missing information were excluded.

A ten-fold cross-validation was conducted to evaluate the performance of our predictive model, where a subset of 10% observations were randomly excluded from the training set and were instead used as an evaluation (test) set. We assessed the accuracy of the model predictions using three different performance metrics, namely, the area under receiver operating characteristic curve (AUROC), the area under precision recall curve (AUPRC), and the model’s confusion matrix. Our deep neural network-based predictive model achieves an AUROC score of 0.970 (±0.001) and an AUPRC score of 0.859 (±0.007). In order to obtain a confusion matrix, we used a threshold that corresponds to the 79.7 percentile of all predicted values to obtain dichotomous predictive results, as patients did not show up 20.3% of appointments in the original set. For context, our logistic regression models achieved an AUROC score of 0.936 (±0.002) and an AUPRC score of 0.855 (±0.006).

“No-shows” at medical appointments cost a significant amount of resources, while handling false alerts (i.e., the case of predicted no-shows but real shows) requires relatively less effort. The confusion matrix using this threshold is generated as shown in Table 2. This method gives precision of 80.7%, recall of 83.0%, and F1 score of 0.82, and our logistic regression method gives precision of 78.4%, recall of 80.2%, and F1 score of 0.79. Both methods outperformed the persistence baseline (precision of 78.9%, recall of 77.2%, and F1 score of 0.78) that consists of taking the previous appointment status as prediction of future behavior. It is worth pointing out that though performance of the neural network-based model is statistically higher than the logistic regression based model, the margin is very small.

**Table 2:** Confusion matrix of neural network model trained and tested on only appointment records without missing information (mean and 90% confidence interval over 10-fold cross-validation are shown).

	Real shows	Real no-shows
Predicted shows	21441.0 [21405.5,21469.1]	1001.0 [969.9,1033.1]
Predicted no-shows	1172.8 [1148.7,1204.2]	4905.7 [4871.8,4937.4]
Confusion matrix (row sum ratio/confidence interval over 10-fold cross-validation).		
Predicted shows	0.96 [0.95,0.96]	0.04 [0.04,0.05]
Predicted no-shows	0.19 [0.19,0.20]	0.81 [0.81,0.81]

**Fig. 2:**
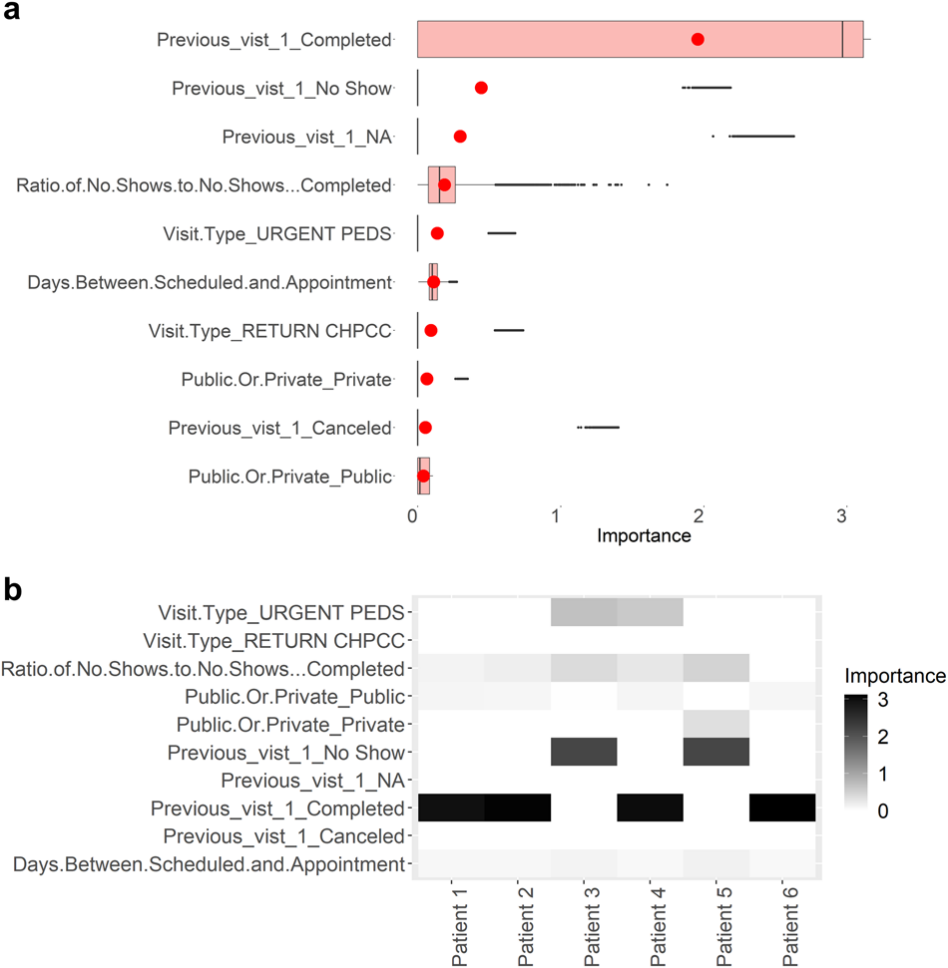
Feature importance in predicting no-shows. **a** Importance of each feature in predicting no-shows, where the top 10 features are shown. The red circle is the mean value of each group. In all boxplots in this study, centreline represents median, bounds of box represent first and third quantiles, and whiskers represent minimum and maximum values excluding outliers. **b** Examples of the importance of each feature in predicting no-shows in different visits of patients. Examples of six visits are shown.

One important factor to consider when designing a predictive model in healthcare is its interpretability. Clinicians, administrators, and patients should be able to understand how and why the predictions are made. To facilitate model interpretability, we calculate the average importance of each feature in the prediction achieved by the neural network model (refer to Fig. 2a). Additionally, it is possible to calculate the importance of each feature in prediction risk of no-show of each appointment. An example is illustrated in Fig. 2b.

### Performance of no-show predictions with missing information of patients

Data missingness is common in various medical datasets and environments. As mentioned in the previous section, 77% of the patients’ records in the study cohort have at least one missing item. Seven features, including “Status of previous visits”, “Mother’s education level”,”Public or private insurance”, “Insurance plan”, “Payor”, “Language“, “Race“, and”Clinical primary care provider’s name“, are missing in at least one record. To address this, first, we focus on adjusting our machine learning-based no-show prediction algorithm so that patients’ records with missing items are accommodated for both training and prediction stages using *missingness indicators*. Second, we explore how to improve the prediction accuracy by identifying patterns of data missingness in the dataset, while including types of missingness into the indicators. Third, we characterize how the missing patterns of different features contribute to the prediction process.

#### Missingness indicators and types of missingness

**Fig. 3:**
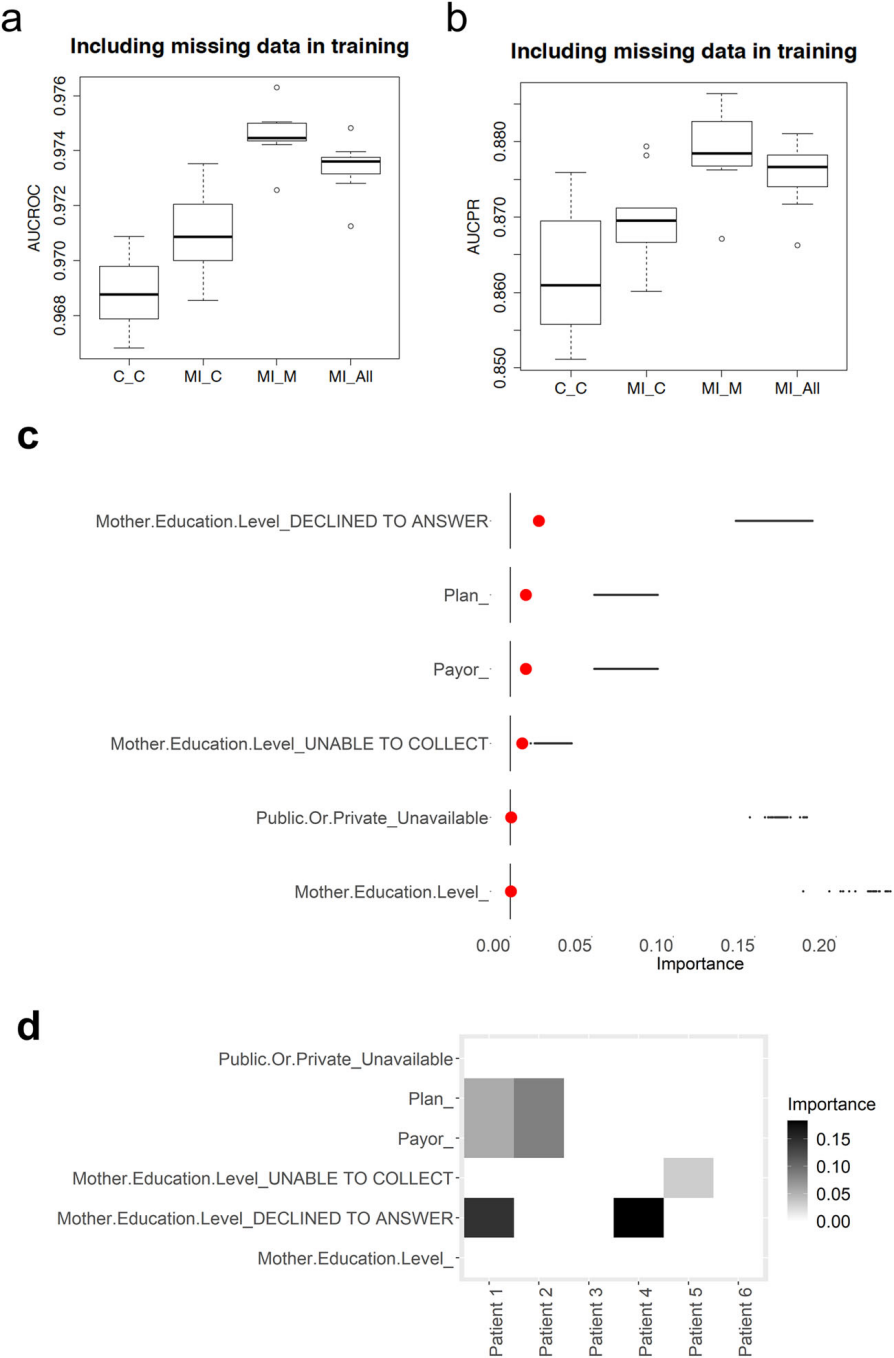
Performance of deep learning models predicting no-shows. **a** AUROC of our deep learning model predicting no-shows for (a) the model trained only on records with complete information and tested on records with complete information, labeled C_C, corresponding to the case of list-wise deletion (b) the model trained on both records with complete and missing information, and tested on records with complete information, labeled MI_C, (c) the model trained on both records with complete and missing information, and tested on records with missing information, labeled MI_M, and (d) the model trained both records with complete and missing information, and tested on records with complete and missing information, labeled MI_All. **b** AUPRC our deep learning model predicting no-shows for the above four experiment settings. **c** Feature importance of each missing information indicator variable, where the top 10 features are shown. **d** Examples of importance of each missing indicator feature in predicting no-shows in different visits of patients. Examples of six visits are shown.

To track the records with missing items in the prediction algorithm, we introduce a series of indicators -binary digits- representing whether each feature is originally missing for the corresponding patient, where 1 and 0 indicate “missing” and “not missing”, respectively. After this adjustment, all the records of 161,822 medical appointments were used either to train or test our resulting machine learning modeling approach. Under this “missingness-type indicator” modeling strategy, the trained machine learning model achieves an average of AUROC score of 0.972 (±0.001) and the average AUPRC score of 0.878 (±0.007) over a ten-fold cross-validation when both records with and without missing information are included in the test sets. For the testing case based on the records with no missing information, the prediction model trained based on both records with and without missing information achieves the AUROC score of 0.973 (±0.002) and the AUPRC score of 0.869 (±0.009), which exhibit statistically superior performance to the model trained based only on data with no missing information (refer to Fig. 3). This improvement is presumably due to the much larger training set used in this modeling strategy. The importance of each missing feature and each type of missingness is shown in Fig. 3c, d, where the race and mother’s educational level appear to be the most important predictors among the features with missing information. Note, in contrast, that naïve list-wise deletion approaches that only consider patients with no missing information for training, are not even able to lead to predictions for unseen patients for whom information is missing.

#### Data imputation does not further improve prediction

Another widely used strategy in machine learning to overcome the sparsity caused by missing data is called data imputation (Efron 1994) Fig. 4. In order to understand whether imputation of missing values in the records will further improve performance of the no-show predicting algorithm, we adopted a Multiple Imputation by Chained Equations (MICE) aided supervised learning-based data imputation technique. Moreover, our empirical findings reveal that although the MICE-aided data imputation technique exhibits potential gains over the case simply using list-wise deletion, it does not show statistically significant improvement compared with the case using the mentioned data missingness indicator (*P*-value > 0.1). Therefore, in all the subsequent sections of this work, only the missingness indicator method is used to handle missing information in patients’ records.

**Fig. 4:**
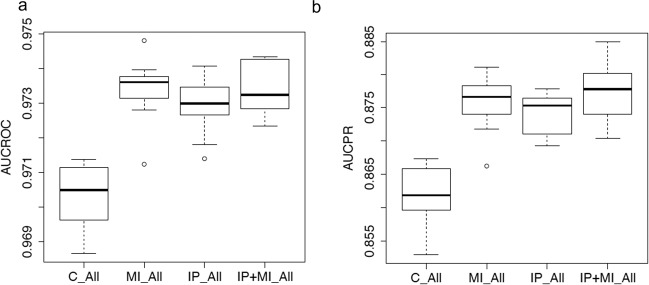
Performance of deep learning models predicting no-shows with different approaches to handle missing information. AUROC and AUPRC of our prediction model trained on the imputed data (IP) and both the imputed data and the missing data indicator (IP + MI). Here, “C” indicates data without missing information; “All” indicates data including all patients with or without missing information; “C_All” represents the model trained only on complete data without missing information and tested on data including all patients; and other models such as “MI_All”, “IP_All”, and “IP + MI_All” are defined in a similar fashion.

### Local weather information improves performance on no-show prediction

Beside information recorded in the medical appointment system, many factors influence whether a patient will miss his/her medical appointment. Among those, we hypothesized that local weather information would be one of the most crucial since bad weather may affect a patient’s transportation means, may affect his/her mental health, may affect his/her health condition. Our results suggest that the inclusion of local weather information can lead to statistically significant improvements on the prediction accuracy of no-shows, even with a small effect.

**Fig. 5:**
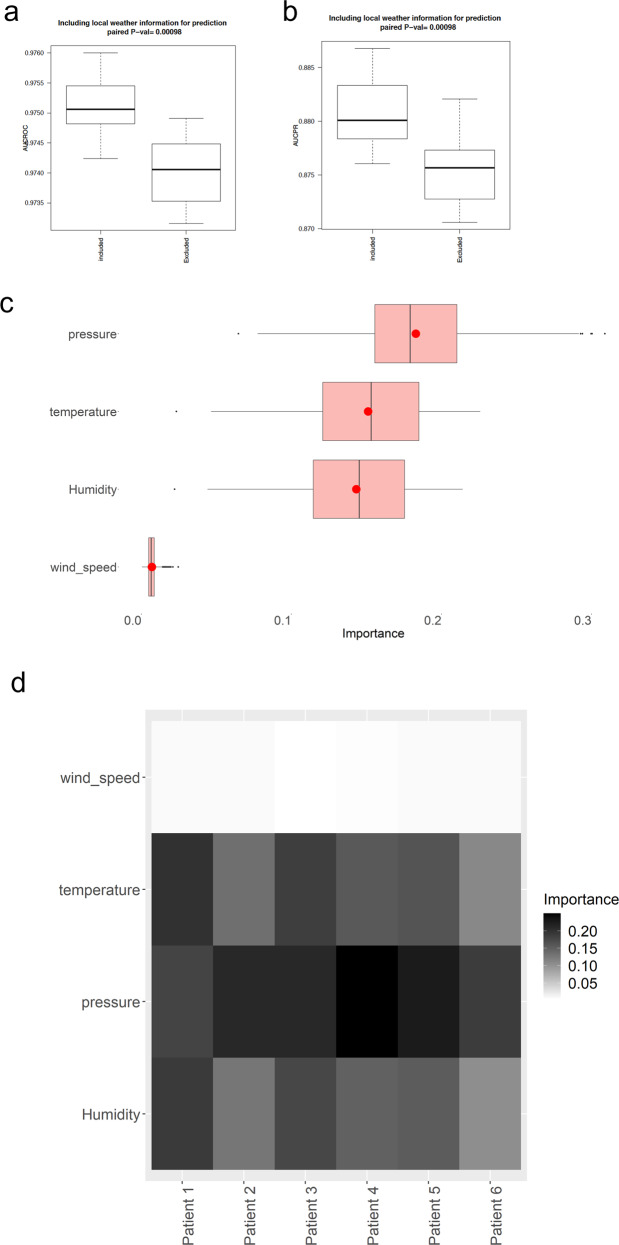
Inclusion of the local weather information as features in predicting no-shows. **a** Inclusion of the local weather information as features in predicting no-shows at medical appointments, where performance is evaluated in terms of AUROC. **b** Inclusion of the local weather information as a predictor in predicting no-shows at medical appointments, where performance is evaluated in terms of AUPRC. **c** Importance of each weather-related feature in predicting no-shows. **d** Examples of importance of each feature in predicting no-shows in different visits of patients.

In our experiments, ambient temperature, wind speed, humidity, and atmospheric pressure in the city where the primary care clinic is located on a day of appointment are included as predictive features. After inclusion of these variables as input features in addition to using missing data imputation/indicator, our model achieved AUROC of 0.975 (±0.001), AUPRC of 0.881 (±0.003), precision of 0.83, recall of 0.83 and F1 score of 0.83 when making prediction on out-of-sample test data that contained both records with and without missing information (Table 3 and Supplementary table 1) Inclusion of such local weather information turns out to show statistically significant improvement on the predictive (refer to Fig. 5). Among the weather-related features, the pressure shows the strongest contribution score, as depicted in Fig. 5c.

**Table 3:** Confusion matrix of neural network method with missing information imputation/indicator and local weather information (mean/confidence interval over 10-fold cross-validation).

	Real shows	Real no-shows
Predicted shows	35789.9 [36738.6, 36842.7]	1832.3 [1800.1,1874.2]
Predicted no-shows	1831.5 [1815.2,1875.6]	8999.9 [7967.7,9029.1]
Confusion matrix (row sum ratio/confidence interval over 10-fold cross-validation)		
Predicted shows	0.95 [0.94,0.95]	0.05 [0.05,0.05]
Predicted no-shows	0.18 [0.18,0.19]	0.83 [0.81,0.83]

#### Potential ways to mitigate no-shows at appointments: associated factors

After identifying which patients have high risks of no-shows, hospitals may want to design strategies to minimize no-shows, which can be generalized into one of two approaches. The first approach is to help the patients with high risks of no-show to show up to their appointments in the clinic. The second approach is to proactively assist patients likely to not show up to reschedule their appointments ahead of time. In general, cancellations/reschedule of appointments result in much less risks to patients’ health compared with plain no-shows because clinicians are able to not only reschedule but also adapt treatment and check-up plans. In this analysis, we do not have enough medical data and information to conduct an analysis on the medical records to search for causal relationship between different actionable items with appointment status. Instead, we aim to identify potential actionable items that are associated with no-shows, which can potentially serve as a starting point for further experiments such as randomized control trials, to identify exact actions that can be taken to reduce no-shows. Our analysis suggests that appointment time, appointment date of week, language of needed services, and choosing a day with desirable weather are potentially actionable items for further study to reduce no-shows. It is worth emphasizing that association analysis presented in this section is derived from the predictive models in previous sections. Languages of the patients are included into the analysis in this section due to potential values in designing interventions, such as providing an interpreter.

**Fig. 6:**
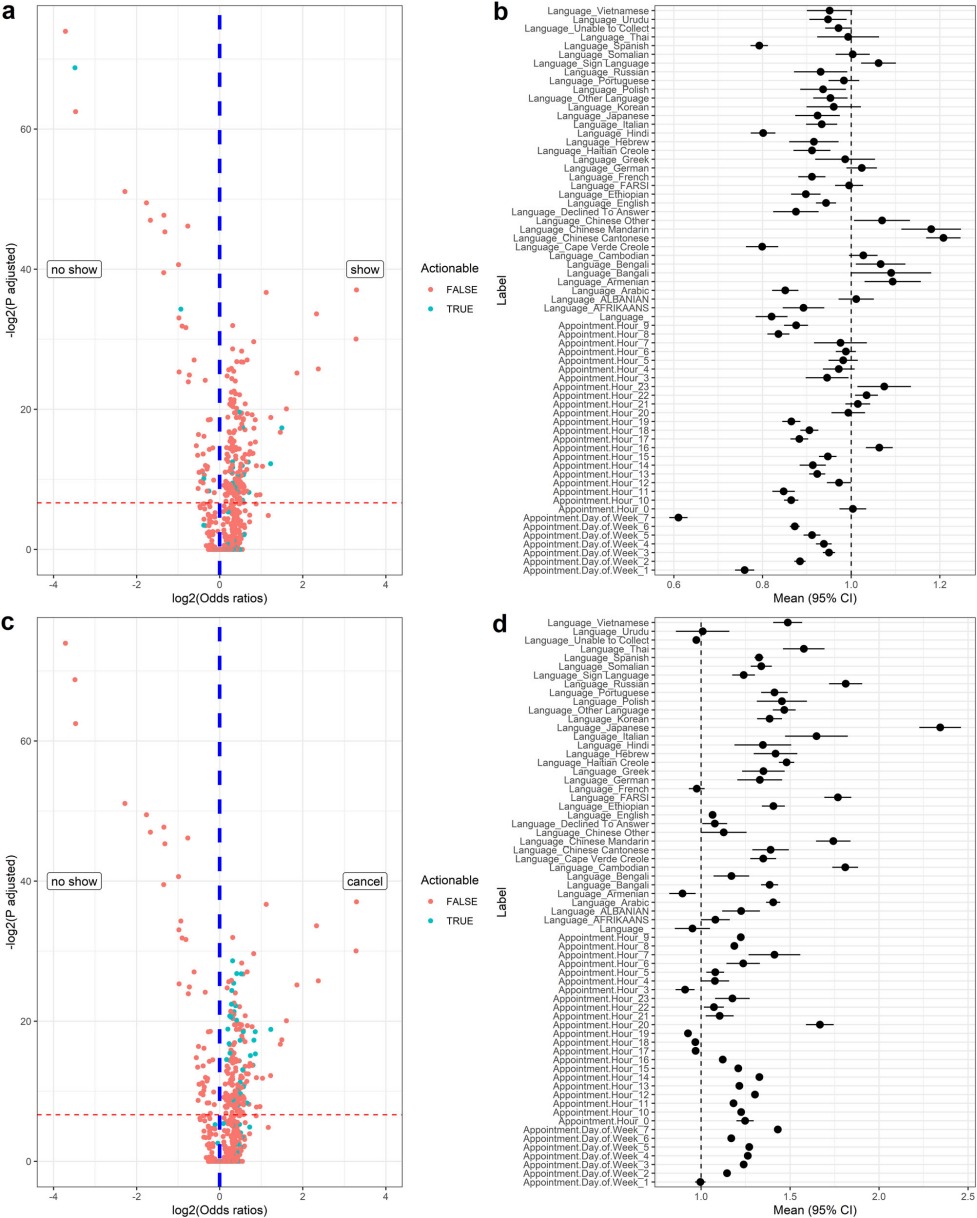
Potential actionable factors associated with show and cancellation at appointments versus no-show. **a** Odds ratio (OR) and adjusted *P* values of association of different features with show (OR > 1, right) versus no-show (OR < 1, left) at appointments. The horizontal red dash line indicates adjusted *P* values = 0.01 and green dots are potentially actionable factors. **b** OR and their confidence intervals of potentially actionable items for show versus no-show. **c** OR and adjusted *P* values of association of different features with cancellation (OR > 1, right) versus no- show (OR < 1, left) at appointments. **d** OR and their confidence intervals of potentially actionable items for cancellation versus no-show.

The first question that we explored is which factors are associated with no-shows. By using a logistic regression model on all available features as input to classify show versus no-show, we are able to estimate effect sizes (i.e., relative odds ratios (ORs)) of each factor. In this analysis, the factor with the highest effective size is “visit type:newborn”, with an odd ratio of 9.8 ± 0.3 (see Fig. 6a, b). However, many of the factors such as “visit type”, “healthcare plan”, and “race” are not actionable or changeable. We thus adopt “appointment day of the week”, “appointment time of the day”, and “language” to be potential actionable items as appointment time often has some flexibility and an interpreter/interpreting system can be provided to people speaking different languages. Among these potentially actionable items, “appointment hours 20:00” (1.67±0.13) and “language: Japanese” (2.34±0.19) have a high OR, towards show-up at appointments. No potential actionable factors show low ORs.

We conduct the same analysis for cancellation versus no-show. “Language_Chinese Cantonese” (1.21±0.06) has a high OR, towards cancellation of appointments; and “appointment day of week 7 (Sunday)” (10.61±0.03) and “Appointment hours: 8:00” (0.83±0.07) show low ORs (see Fig. 6c, d).

Next, we explored the potential causal effects of different local weather conditions on no-show risks so that the appointment can be rescheduled to another day with better weather conditions to minimize the risks of no-show due to the fact that weather conditions can be obtained beforehand from weather forecasts. In general, the causal effects indicate that if a certain action, such as scheduling a medical appointment on a sunny day, is taken, then the outcome (i.e., risks of no-show) will change accordingly, which is not necessarily in the correlation relationship, as in the predictive model. In other words, correlation is not the same as causality.

**Fig. 7:**
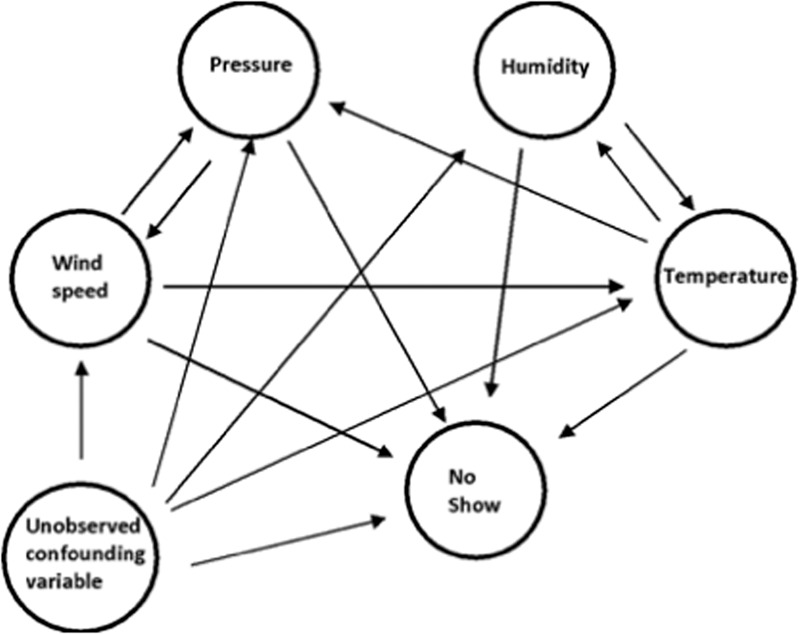
Illustration of the estimated causal relation among weather-related features and no-show at medical appointments along with the graphical model. Further experiments and analysis will be needed to obtain the real causal graph.

Using the graphical model in Fig. 7 and the backdoor linear regression method, we estimate causal effects of the four local weather variables on no-shows. Both temperature and humidity exhibit statistically significant estimated causal effects of −0.0134 with *P* value = 0.001 and *P* value = 0.002, respectively. The causal effect of pressure is not statistically significant even with a strong correlation. Wind speed has the largest estimated causal effect of 0.045 with *P* value = 0.003 (refer to Supplementary Fig. 5). Generally, these results imply that in the city where the hospital of interest is located in, scheduling the appointment on a day with a relative higher temperature, higher humidity, and lower wind speed level will enable us to reduce the risks of no-show at the pediatric primary care clinics. This is intuitively understandable as the city of Boston is in a cold region of the country. It should be noted that it is impractical to infer causal relationships from retrospective data. The estimated causal relationships in this section suggest potentials of taking weather into consideration when designing intervention methods for no shows and serve as a starting point for further exploration. More experiments and studies will be needed to discover the real causal graph.

## Discussion

We developed a personalized and interpretable methodology to predict a patients’ risk of no-show in a major pediatric primary care clinic. We used a data imputation strategy, based on a collection of missingness-type indicators, to address the frequent missing information in patients’ records and showed that inclusion of patients’ records with missing information significantly improves the predictive accuracy, when compared to a baseline approach that can only be trained and assessed on patients for whom complete information is available (only 23% in the dataset). In addition, our analysis suggested that inclusion of local weather information into predicting features improves model accuracy. Finally, we identified potentially actionable ways for further studies to explore how to reduce patients’ no-shows.

Both our neural network-based and logistic regression based binary classification models outperformed a persistence baseline approach (that only uses the most recent information on no-show behavior of a patient) (See Supplementary Materials for details). Analysis on feature importance of our models suggest that the history of patients’ no-show records is the most important predictor, which is consistent with prior studies^25^. Among the local weather-related variables found to be associated with no-shows, atmospheric pressure was shown to be the most important predictor.

Our no-show prediction method may potentially be informative when identifying appropriate interventions to reduce no-shows. Specifically, the output of our models can be used to identify patients for whom a text, email, or call (reminding them of their scheduled appointment) may reduce their likelihood of no-show. It may also be useful to calculate the optimal frequency at which reminders should be sent for each patient and may help better allocate free transportation resources when needed. In addition, our no-show prediction model could also be used to optimize scheduling systems and minimize patients’ waiting time. We acknowledge that clinics with limited resources, the interventions mentioned above may not always be available. With this limitation in mind, we explored potential actionable items that could be implemented in the majority of clinics. Our results suggest that choosing the day of the week and time of day that would be easier for patients and their parents to come to their medical appointments, and moreover using a language service and choosing a day with likely nicer weather would help reduce no-shows. These effects were shown to be the most related to working hours of parents, traffic in the city, and potentially school schedules of the patients. Atmospheric pressure, frequently an indicator of potential changes in precipitation, was shown to be the most important weather-related predictor of no-shows. The fact that other variables such as wind speed, temperature, and humidity have statistically significant causal effects on no-show risks can be leveraged as a key reference when a way of interventions for high-risk patients is suggested. It is worth re-emphasizing that due to the lack of data and information, potentially actionable items identified in this study would need to be subject to further validation by experiments such as randomized control trials in clinical settings.

In terms of fairness, the model trained with no race or race proxy features, as input, shows minimal predictive performance differences across race groups when tested on unseen patients (<1% by AUCROC, Supplementary Fig. 4). Therefore, we decided that there was no need to pursue additional methodologies to address fairness issues in this study. These minimal performance differences may emerge due to differences in sample sizes, both in the training and testing sets.

Research conducted in this article has limitations. First of all, due to limited data access, we only have patients’ records from a single primary care clinic. This limits generalizability of both the trained algorithm and the scientific conclusion. Secondly, we only have access to a small portion of records of the patients for no-show prediction. Access to more records of a patient will lead to further improvement of the prediction accuracy. Thirdly, in this study, we identified several potential interventions to reduce no-shows. In certain institutions, data on medical appointments such as whether reminder messages and/or emails were sent could be used to evaluate more effective interventions, which were not available in this study. In addition, although our neural network model shows statistically significant improvement compared with the logistic regression model, the effect size is small; our speculation is that this small effect size is caused by a small feature space due to limited data access. If other patient information such as diagnosis and prescription could be incorporated into features, we suspect the performance of our neural network model may be further enhanced. The importance of features showed strong correlation between the neural network and logistic regression models, where one of the top predictive features in both models is the history of appointments (Supplementary Fig. 1, Table 2, and Table 3). These suggest that a neural network model approach may not be justified in practice due to its significant higher computational cost when compared to a logistic model.

A potential avenue of future research could be the development of no-show predictive models for an array of patients visiting different healthcare providers. We suspect that adding information on the different medical problems affecting patients may improve predictive performance. The no-show prediction method developed in this article is easily generalizable and could possibly be implemented in hospitals making use of the SMART-on-FHIR systems that retrieves individual’s historical information when making an appointment^26^. In addition, CDS Hooks can be used to further integrate the predictive algorithm into clinical workshops, where the predictive algorithm can be continuously trained as new data become available and are shared among multiple healthcare providers^27^.

## Methods

### Data processing

The data of medical appointments were collected from the outpatient clinic of a major academic pediatric hospital for a quality improvement purpose. The data contain appointments of all departments and visit types of the clinic and include the names of the healthcare providers. All categorical data types are converted to multiple binary variables, where 1 and 0 indicate the presence and absence, respectively, of each feature. For example, the feature “day of the week of the appointment” originally has values from 1 to 7, representing Sunday to Monday of the week. In our analysis, we converted the feature to seven separate binary indicators with 1 indicating that the appointment is made on that day of the week. All numerical features were normalized so that they lie between 0 and 1. The labels are binary digits, where 1 and 0 denote no-shows and shows, respectively, at the corresponding appointment. A single patient may have more than one records due to multiple appointments. The vast majority of the patients are children and often come to the appointments with their parents or caregivers. A patient’s appointment status is one of the following: “completed”, “canceled” or “no show”. All patients have an appointment status recorded. We conducted a binary classification to predict if a patient will have a “no show”. The hospital providing data used in this study is a pediatric academic medical centre. Therefore, most of the patients are children (⩽age 18). Only in some rare cases (583 patients or 3% in our cohort), older patients with certain medical conditions for which this hospital has unique expertise on continue to seek healthcare in the same institution. Due to this special situation and to avoid any confusion, we excluded all patients above age 18 from our cohort in this study.

### Deep neural network model and its training

Artificial neural networks are used as a primary machine learning model for predicting no-shows. The model is constructed in the Keras 2.0 environment using TensorFlow 1.7 as backend. The cross-entropy is used as a loss function, and Adam is used as a gradient descent method with default settings. The model consists of 3 hidden layers with 32,256, 12 neural units. The output layer has 1 neuron. The activation function used for the hidden layers and the output layer is “ReLu” and “Sigmoid”, respectively. In each cycle of cross-validation, the data are randomly split into the training set (75%) and the testing set (25%). The model is first trained for 20 epochs using all the training data. Hyperparameters were determined by using validation set not including in testing. After hyperparameters are determined, validation and training sets are combined to train the model. When training the three-class classification algorithm in Supplementary materials, “softmax” was used as an activation function of the last layer and the categorical cross-entropy was used as a loss function.

### Data missingness and missingness indicator

A common practice to deal with such missing data is to adopt the list-wise deletion (or equivalently, complete-case analysis) method in which the records with missing items are naively deleted^28^. However, this list-wise deletion approach not only introduces a bias into the dataset, as medical data are rarely missing completely at random (MCAR) but rather missing at random (MAR) and/or missing not at random (MNAR). Note that MAR occurs when the missingness depends on information that we have already observed while MNAR occurs when the probability that data are missing depends on the unobserved data^18^. This naive approach to dealing with missing data also has some limitations due to the fact that it typically prevents the resulting model to prospectively predict an outcome for a new (unseen) patient with a record that contains missing information. In addition, data missingness may cause severe data sparsity problems that may lead to poor prediction.

We used binary missingness indicators to handle features with missing values where 1 and 0 indicate “missing” and “not missing”, respectively. We also note that there exist multiple types of “missingness” for a given feature; If available, then different types of data missingness are recorded by a distinct collection of “missingness indicators” for the same feature. For example, for Mother’s educational level, there are three different missing indicators. If the patient or the parents declined to answer the question, the” Mother’s educational level_DECLINED TO ANSWER“ is marked as 1 and other two are marked as 0. If due to some operational reasons the answer cannot be collected,” Mother’s educational level_UNABLE TO COLLECT’“ is marked as 1. If the information is missing due to any other reasons,” Mother’s educational level_UNAVAILABLE” is marked as 1.

### Supervised learning-based data imputation

We attempted to conduct data imputation. Imputation methods that have been commonly adopted in the literature are generally categorized into: i) single imputation, which generates a single imputed value for each missing data point, and ii) multiple imputation, which draws imputed values multiple times according to a probability distribution. In this study, we adopt Multiple Imputation by Chained Equations (MICE), which accounts for the uncertainty in imputation to impute the missing items in the medical appointment dataset^29^. In this study, we used an artificial neural network model consisting of 3 hidden layers to impute each missing variable as binary classification tasks using available variables as input. Our results show that an inclusion of records with imputed missing information in training leads to the performance improvement compared with the case only using complete records (i.e., the case using list-wise deletion) but no statistically significant improvement compared with the missing indicator method is observed. Therefore, data imputation was not conducted in analyses in this study.

### Baseline methods

Our persistence baseline method uses the appointment status of the latest visit as prediction for the next visit. If information on the previous visit of a patient was not available, a random status among show, cancel, or no-show is assigned to the patient using P(show) = 1 − (no-show rate of the patient) − (average population rate of cancellation), *P*(no-show) = no-show rate of the patient and P(cancel) = average population rate of cancellation as the probability. Logistic regression models were trained without regularization and optimized using gradient descent for efficiency. Multinomial NaÏve Bayes was used for a NaÏve Bayes model.

### Performance metrics

The ROC curve is produced by plotting the true positive rate (TPR) (or sensitivity) and the false positive rate (FPR) (or 1-specificity) at different thresholds ranging from 0 to 1. The prediction scores (i.e., the predicted probabilities of no-shows) are compared at each threshold. The AUROC is calculated along with values closer to 1 indicating good quality prediction and values closer to 0.5 indicating bad quality prediction. This principle is also applied for the AUPRC in a similar manner.

### Interpretation of our prediction model

In order to interpret how our deep neural network makes the predictions, DeepLIFT was applied to the model as DeepLIFT explains the difference (i.e., the error) between the predicted output and some”reference” output^30,31^. The method estimates the importance of each feature in prediction for each input by calculating the difference by comparing with the reference point.

### Local weather data

Local weather data were obtained from the OpenWeather website (url: https://openweathermap.org/). Temperature (in Kelvin), pressure (in hectopascal pressure units), humidity (in %), and wind speed (in m/s) were aggregated as each day.

### Causal inference

The first step of conducting the causal inference is to build a causal graph (refer to Fig. 7). We assume that all the four local weather variables (i.e., weather-related features) could potentially have causal effects on medical appointment no-shows and moreover there exist potentially unobserved confounding factors. Temperature affects air pressure due to the velocity of air molecules as warm air can hold more water vapor than cold air. On the other hand, since humidity affects heat conductivity of air, humidity and temperature are assumed to have a causal relation with each other in the graph. Wind speed affects the rate of cooling and is caused by different air pressures.

The causal graph is generated manually. Causal effects were calculated using the backdoor linear regression method by assuming conditioning on confounding factors^32^. “DoWhy”, a causal inference library in the Python 3.6 environment, was used in this study. It is worth pointing out that the value of causal effects from observed data is only seen as estimates of the real causal effects, which can usually be obtained from experiments.

### Reporting summary

Further information on research design is available in the Nature Research Reporting Summary linked to this article.

## Supplementary information

supplementary materials

Reporting Summary

Supplementary list 1

## Data Availability

The data that support the findings of this study are available on request from the corresponding author (M.S.). The data are not publicly available as individual level healthcare data are protected by privacy laws.

## Code availability

Machine learning model codes for this study are available at https://github.com/kaiyuanmifen/medicalappointment. The imputation pipeline and machine learning models are constructed in the Keras 2.0 environment using TensorFlow 1.7 as backend using Python 3.6.
