# Supplementary material for: Machine learning approaches to predicting no-shows in pediatric medical appointment
Source: NPJ Digit Med. 2022 Apr 20;5:50. doi: 10.1038/s41746-022-00594-w (PMC9021231; doi:10.1038/s41746-022-00594-w)
Supplement: Supplementary file 1 — supplementary materials [file 41746_2022_594_MOESM1_ESM.docx]

# Supplementary information


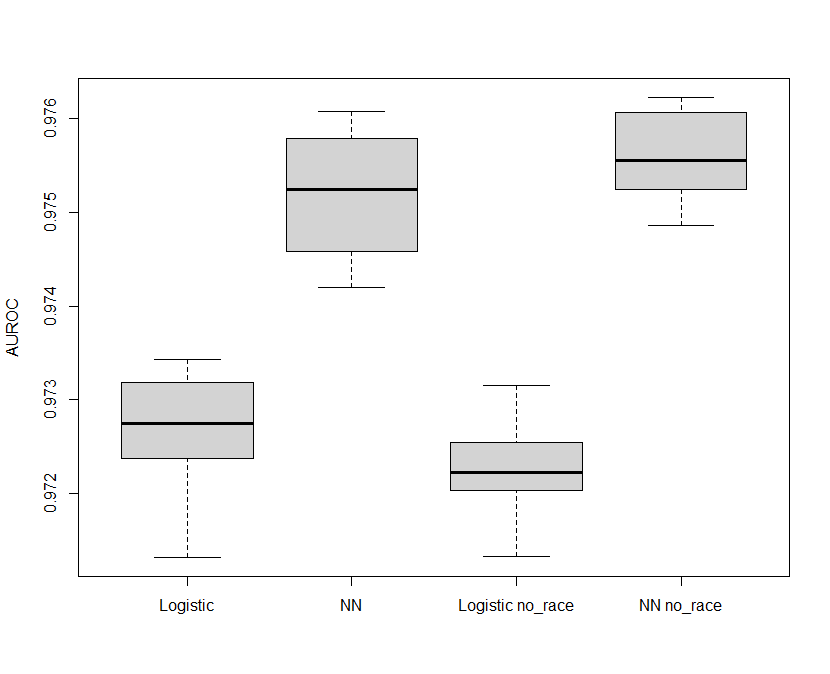


Supplementary Figure 1. Exclusion of race and race proxy features do not cause statistically significant reduction in the model performance measured by AUROC. P-values for both logistic regression and neural network (NN) are > 0.05.


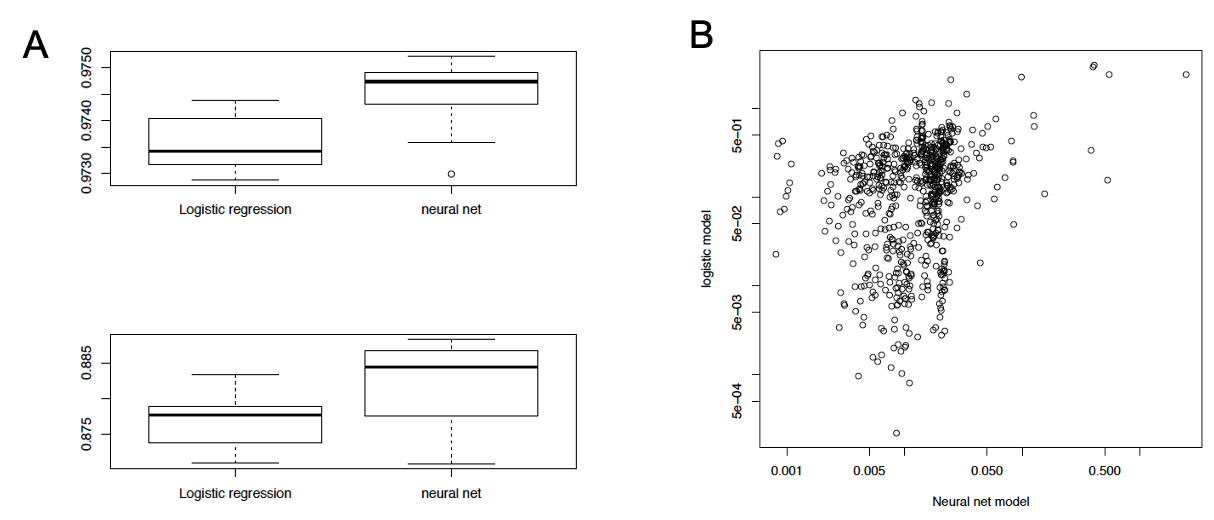


Supplementary Figure 2. (A) Logistic regression versus neural network models with data missing type plus weather information. Upper and lower panels are AUROC and AUPRC, respectively. (B) Importance (weights) of different features in predicting no-show. Y axis: logistic regression model. X axis: neural network model. Correlation=0.46. P<0.001


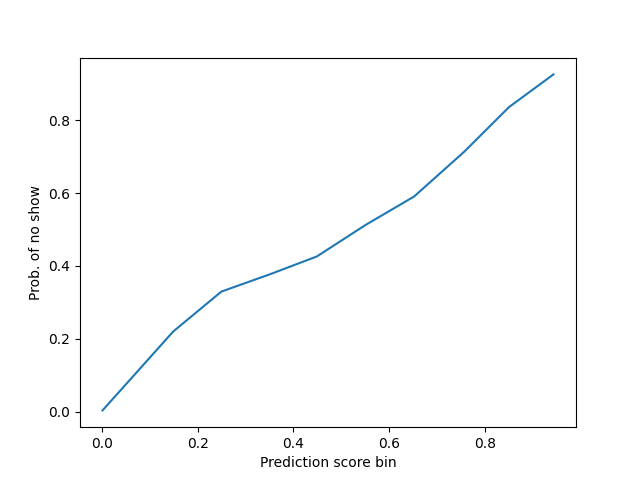


Supplementary figure 3. Calibration curve of the neural network model when the number of bins is 10.

**
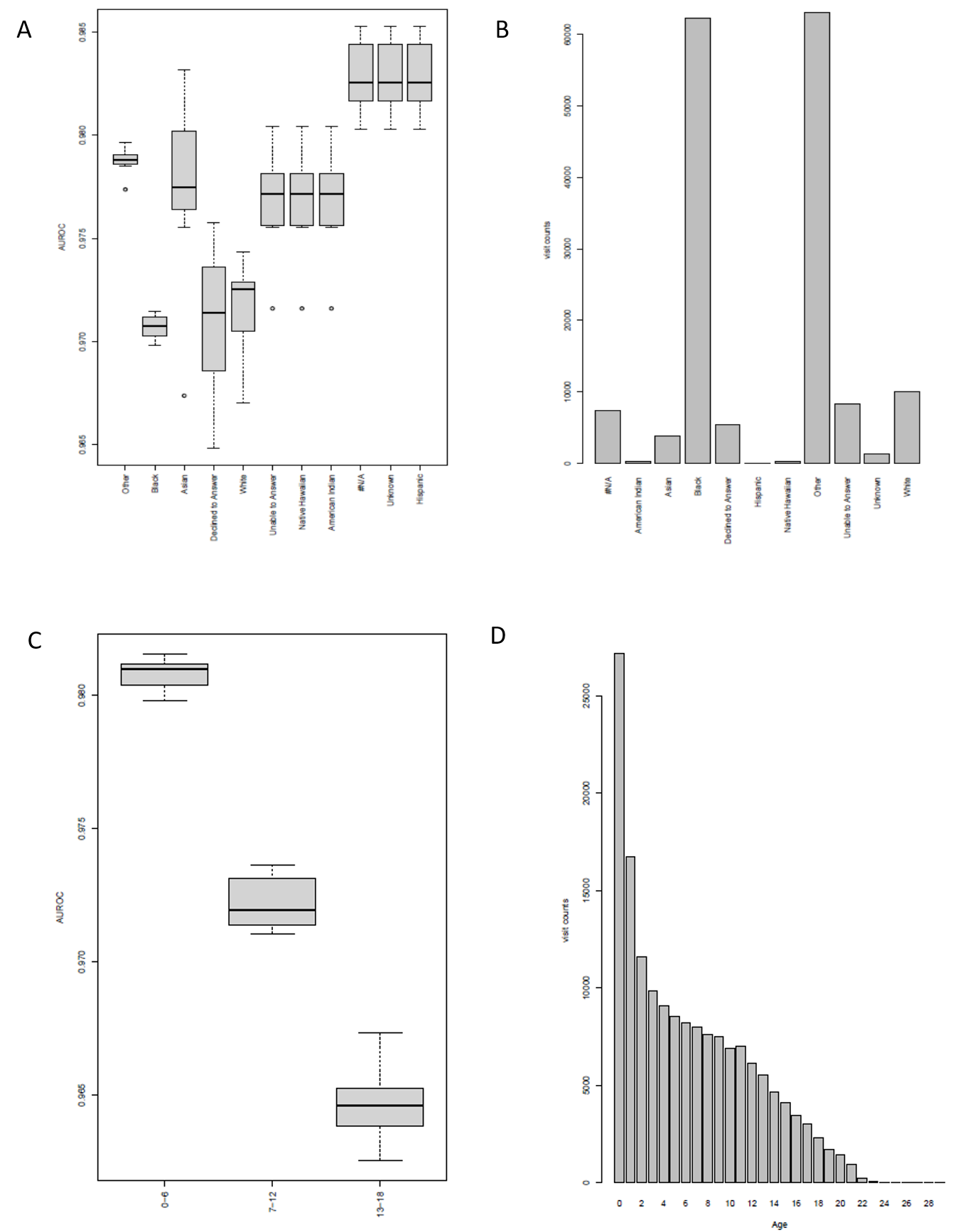
**

Supplementary figure 4. No show (NN) prediction model performance and number of visits in the data set by race (A, B) and age (B,D) groups. Y axis is AUROC in A and C. Some race groups have a very small number of patients, therefore the range of AUROC looks uniform.


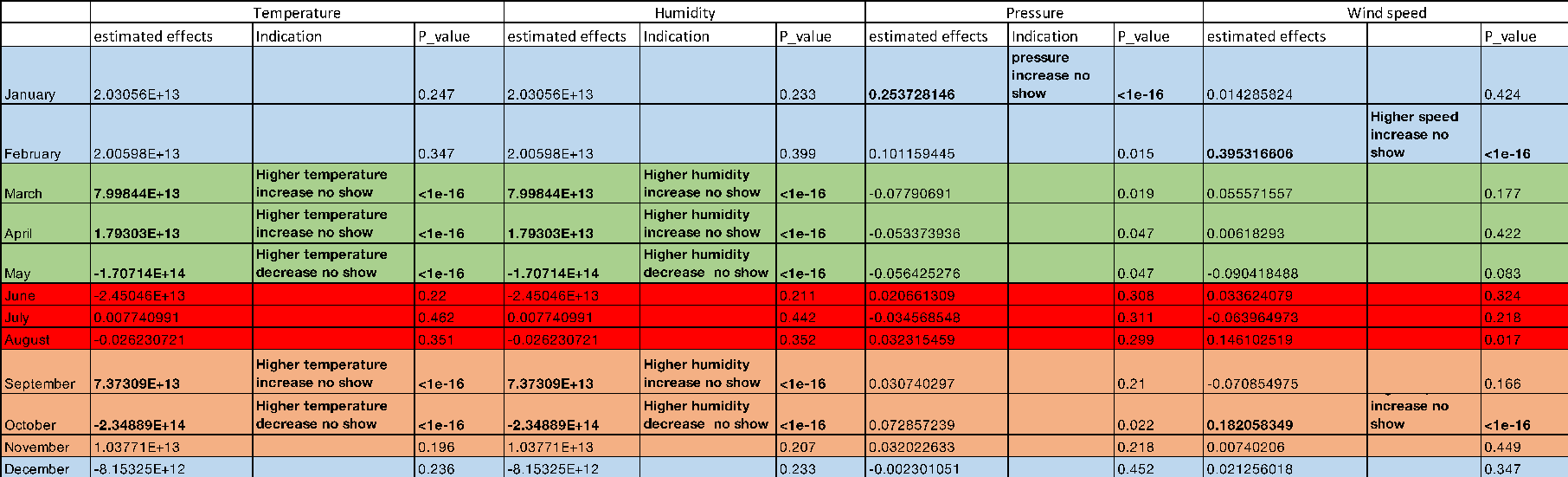


Supplementary figure 5. Estimated causal effects of weather-related features on no-shows in different months of the year, where different colors represent different seasons

**Supplementary table 1. Precision, recall, and F1 score of baseline methods and our approach**

|  | **Baseline (persistence)** | **Logistic regression (history only)** | **Logistic regression (with imputation/indicator and include local weather information)** | **Neural**  **network-based method (with missing information imputation/indicator include local weather information)** |
| --- | --- | --- | --- | --- |
| **Precision** | 0.64 | 0.80 | 0.81 | 0.83 |
| **Recall** | 0.98 | 0.81 | 0.82 | 0.83 |
| **F1** | 0.78 | 0.80 | 0.81 | 0.83 |

**Supplementary table 2. top 20 most important features in predicting no-shows in our neural network model**

| Feature | Mean score |
| --- | --- |
| Previous_vist_1_Completed | 2.44390 |
| Previous_vist_1_No Show | 0.54366 |
| Days.Between.Scheduled.and.Appointment | 0.52584 |
| Ratio.of.No.Shows.to.No.Shows...Completed | 0.40645 |
| Previous_vist_1_NA | 0.39766 |
| pressure | 0.38228 |
| Language_English | 0.15424 |
| Visit.Type_URGENT PEDS | 0.12553 |
| Visit.Type_RETURN CHPCC | 0.12444 |
| Previous_vist_1_Canceled | 0.09797 |
| wind_speed | 0.08404 |
| temperature | 0.08348 |
| Humidity | 0.08341 |
| weather_description_mist | 0.08051 |
| Public.Or.Private_Public | 0.07073 |
| Race_Other | 0.06116 |
| Appointment.Date.Time..Month.of.Year._April | 0.05953 |
| Race_Black or African American | 0.05747 |
| weather_description_light rain | 0.05368 |
| weather_description_few clouds | 0.05063 |

**Supplementary table 3. top 20 most important features in predicting no-shows in our logistic regression model**

| Feature | Importance score |
| --- | --- |
| Previous_vist_1_Completed | 2.42568513 |
| Previous_vist_1_No Show | 2.42684533 |
| Days.Between.Scheduled.and.Appointment | 0.153721411 |
| Ratio.of.No.Shows.to.No.Shows...Completed | 3.08709121 |
| Previous_vist_1_NA | 2.95971436 |
| pressure | 0.335858268 |
| Language_English | 0.108464511 |
| Visit.Type_URGENT PEDS | 0.63015907 |
| Visit.Type_RETURN CHPCC | 0.835925781 |
| Previous_vist_1_Canceled | 2.27176397 |
| wind_speed | 0.049340496 |
| temperature | 0.257498967 |
| Humidity | 0.243839736 |
| weather_description_mist | 0.424207526 |
| Public.Or.Private_Public | 0.165831679 |
| Race_Other | 0.129652853 |
| Appointment.Date.Time..Month.of.Year._April | 0.757974815 |
| Race_Black or African American | 0.095366389 |
| weather_description_light rain | 0.367293397 |
| weather_description_few clouds | 0.63036262 |

Our predictive models achieved a high level of accuracy and perform better than the persistence baseline and logistic regression method. To illustrate the full spectrum of the data, three-class classification results are shown in supplementary table 3, 4, and 5, where our neural network model still shows improvement compared with logistic regression and NaÏve Bayes baseline models.

Supplementary table 4. Performance of the NaÏve Bayes model in three-class classification

|  | **precision** | **recall** | **f1-score** |
| --- | --- | --- | --- |
| **show** | 0.95 [0.95,0.95] | 0.92 [0.92,0.92] | 0.93 [0.93,0.93] |
| **cancelled** | 0.27 [0.26,0.29] | 0.11 [0.11,0.12] | 0.16 [0.15,0.17] |
| **no show** | 0.70 [0.70,0.70] | 0.87 [0.86,0.87] | 0.77 [0.77,0.78] |

Supplementary table 4. Performance of the baseline method in three-class classification

|  | **precision** | **recall** | **f1-score** |
| --- | --- | --- | --- |
| **show** | 1.00 [1.00,1.00] | 0.85 [0.85,0.85] | 0.92 [0.92,0.92] |
| **cancelled** | 0.25 [0.25,0.25] | 0.28 [0.27,0.28] | 0.26 [0.26,0.26] |
| **no show** | 0.64 [0.64,0.64] | 0.98 [0.98,0.99] | 0.78 [0.78,0.78] |

Supplementary table 4. Performance of the neural network model in three-class classification

|  | **precision** | **recall** | **f1-score** |
| --- | --- | --- | --- |
| **show** | 1.00 [1.00,1.00] | 0.89 [0.88,0.89] | 0.94 [0.94,0.94] |
| **cancelled** | 0.33 [0.33,0.34] | 0.30 [0.28,0.31] | 0.31 [0.31,0.32] |
| **no show** | 0.69 [0.69,0.70] | 0.99 [0.98,0.99] | 0.81 [0.81,0.82] |

Supplementary List 1. Full list of features available in a patient’s record for this study
